# Motion Intention Prediction for Lumbar Exoskeletons Based on Attention-Enhanced sEMG Inference

**DOI:** 10.3390/biomimetics10090556

**Published:** 2025-08-22

**Authors:** Mingming Wang, Linsen Xu, Zhihuan Wang, Qi Zhu, Tao Wu

**Affiliations:** 1 School of Mechanical and Electrical Engineering, Hohai University, Changzhou 213022, China; 231319010023@hhu.edu.cn (M.W.); 220219030003@hhu.edu.cn (Z.W.); 2 Suzhou Research Institute, Hohai University, Suzhou 215000, China; 3 Changzhou Power Supply Branch, State Grid Jiangsu Electric Power Co., Ltd., Changzhou 230026, China; 15351981698@163.com; 4 Wuhan Second Ship Design Institute, Wuhan 430205, China; thewutao@163.com

**Keywords:** lumbar spine assisted robot, surface electromyographic signals, multimodal information fusion, VSPAM

## Abstract

Exoskeleton robots function as augmentation systems that establish mechanical couplings with the human body, substantially enhancing the wearer’s biomechanical capabilities through assistive torques. We introduce a lumbar spine-assisted exoskeleton design based on Variable-Stiffness Pneumatic Artificial Muscles (VSPAM) and develop a dynamic adaptation mechanism bridging the pneumatic drive module with human kinematic intent to facilitate human–robot cooperative control. For kinematic intent resolution, we propose a multimodal fusion architecture integrating the VGG16 convolutional network with Long Short-Term Memory (LSTM) networks. By incorporating self-attention mechanisms, we construct a fine-grained relational inference module that leverages multi-head attention weight matrices to capture global spatio-temporal feature dependencies, overcoming local feature constraints inherent in traditional algorithms. We further employ cross-attention mechanisms to achieve deep fusion of visual and kinematic features, establishing aligned intermodal correspondence to mitigate unimodal perception limitations. Experimental validation demonstrates 96.1% ± 1.2% motion classification accuracy, offering a novel technical solution for rehabilitation robotics and industrial assistance.

## 1. Introduction

In recent years, lumbar spine injury has emerged as a significant global public health challenge due to its high incidence rate and associated disability. As the mechanical core of human spinal dynamics, the lumbar spine critically enables trunk stabilization through multi-degree-of-freedom coupled motions. Its biomechanical properties directly govern spinal functional compensation and injury risk potential [1]. In the field of low back rehabilitation, assistive exoskeletons based on motion intention recognition demonstrate significant clinical value. However, existing devices exhibit substantial limitations in compliance adaptation and neuromuscular synergy control.

In 2023, DING et al. [2] proposed a new type of passive exoskeleton design, using a spring-cable-differential mechanism as a torque generator to drive the two hip joints. It provides assistance based on different human states, offering auxiliary torque during lifting and low resistance during walking. It can automatically distinguish between lifting and walking. Regarding the current research on passive lower back exoskeletons, which focuses on reducing the activation of lumbar muscles by providing extensor torque, MOON et al. [3] proposed a new type of passive lower back exoskeleton that can provide extensor torque and lumbar traction force. The lumbar traction force can reduce compressive force, and a helical spring is used for assistance during bending and flexion.

In recent decades, biological signals have gained prominence in robot control. Typical signals include Electroencephalogram (EEG) and Electromyogram (EMG). Surface EMG signals, widely employed in rehabilitation therapy, confront three principal technical bottlenecks: weak signal characteristics (50–2000 μV amplitude) causing susceptibility to electrocardiographic (ECG) coupling interference, Power Line Interference (PLI), and motion artifacts; individual anatomical variations (differences in subcutaneous fat thickness and muscle fiber arrangement) inducing signal characteristic drift; and time-varying dynamics stemming from electrode-skin interface impedance changes in dynamic scenarios. To address these challenges, current research employs a three-stage processing paradigm: front-end contact impedance suppression via Ag/AgCl gel electrode and conductive media optimization; middle-end noise suppression and signal enhancement using improved wavelet packet transforms; and back-end construction of Domain-Adversarial Neural Networks (DANN) for cross-individual feature migration learning.

At the feature engineering level, traditional methods rely on the manual design of feature sets (time domain: Mean Absolute Value (MAV), Root Mean Square (RMS), Waveform Length (WL), Zero Crossing Rate (ZC)) [4,5,6,7]; frequency domain: Maximum Frequency Density (MDF), Median Power Frequency (MPF) [4,5,8,9]; Time-frequency analysis domain: Hilbert-Huang Transform (HHT) marginal spectral energy [10,11,12,13]), but non-smooth signal properties lead to limited feature separability. Deep learning achieves direct mapping from raw signals to action semantics by constructing end-to-end models (e.g., 58-dimensional HHT statistics, continuous wavelet time-frequency coefficients). A multimodal fusion strategy (surface Electromyogram (sEMG) in concert with Inertial Measurement Unit (IMU) data) improves dynamic gesture recognition accuracy by 12.7%, and t-SNE visualization confirms that the wrist electrode array has optimal interclass separability in seven types of gesture classification.

Typical research advances include Xu et al. [14] leveraging Convolutional Neural Network (CNN) to process sEMG energy kernel feature maps while synchronously fusing IMU data to enhance system robustness, though IMU signal synchronization remains; Prabhavathy team [15] achieved 98.04% average classification accuracy using a CNN-LSTM dual-stream network; Wei et al. [16]. proposed a lower-limb motion recognition framework integrating adjustable Tunable Q-Factor Wavelet Transform (TQWT) with Kraskov entropy (KrEn), achieving 98.42% average recognition accuracy validated by Linear Discriminant Analysis (LDA) classification. These advances establish foundational technical support for real-time capability, generalization capacity, and individual adaptation in wearable human–computer interaction systems.

To address these technical bottlenecks, this study developed a variable-stiffness lumbar spine-assisted exoskeleton system, with core breakthroughs in three key aspects:

(1). Bionic variable stiffness drives unit innovation: breakthrough of the traditional Pneumatic Artificial Muscle (PAM) single degree of freedom drive limitations.

(2). Optimization of man-machine compatibility structure: Based on the kinematic characteristics of lumbar spine L1–L5 segments, an exoskeleton framework with bionic joint coupling characteristics is developed. Adopting modular stiffness adjustment unit and 3D printing customized strap system, the wearable fit error is <2 mm, which effectively reduces the soft tissue shear stress during movement.

(3). Neuromuscular synergistic control strategy: a two-channel deep feature fusion model (VGG-LSTM) is proposed to extract sEMG signal Gramian Angle Field (GAF) features via a VGG16, which is combined with a LSTM to decode the lumbar flexion-extension-rotation composite motion intention. Experiments show that this model has a classification accuracy of 95%. This significantly improves the reliability of human–computer interaction.

Through tripartite innovation in electromechanical systems, biochemical decoupling, and intelligent algorithms, this study developed an intelligent rehabilitation exoskeleton platform featuring a closed-loop “perception-decision-execution” architecture. This platform delivers an integrated solution for elderly patients with degenerative diseases and chronic lumbar muscle strain, ensuring both biological safety and motor function compensation—establishing a milestone in clinical translation of flexible rehabilitation robotics. Compared to traditional exoskeleton robots, this innovation significantly reduces the user’s muscle load (40% ± 5% reduction), greatly enhances wearing comfort, thereby demonstrating considerable application potential and market value in areas such as protecting worker health, improving work efficiency, and assisting vulnerable groups. This strategy, which maintains the human body’s work contribution ratio at 58%, effectively alleviates fatigue while preserving an essential sense of engagement for the wearer.

## 2. Structural Design and Motion Characterization of Exoskeleton

### 2.1. Overall Design of the Exoskeleton Robot

**Figure 1:**
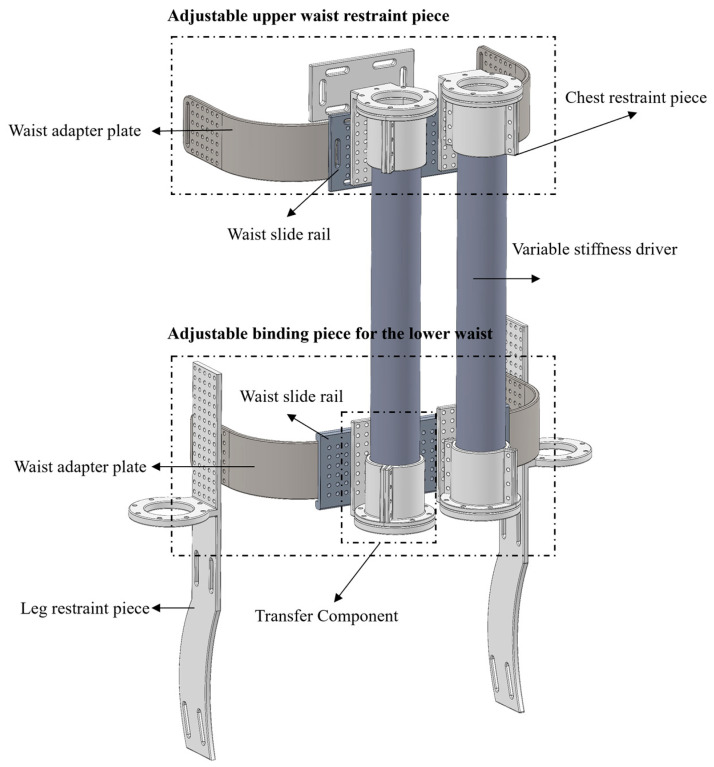
Lumbar spine assisted exoskeleton robot 3D model drawing.

The lumbar spine-assisted exoskeleton robot presented in this study primarily comprises mechanical and pneumatic systems, as shown in Figure 1 including the following: the VSPAM with structurally consistent upper and lower lumbar adjustable restraints; the VSPAM connects between these restraints. Utilizing pneumatic actuation, pressurizing the VSPAM with high-pressure gas induces actuator contraction, thereby generating contractual force.

The lumbar spine-assisted exoskeleton robot designed herein employs two variable stiffness actuators, affixed to both sides of the human body, for rehabilitative and assistive training of the lumbar region. These bilateral variable stiffness actuators can drive the lumbar spine to bend leftward, rightward, forward, and backward, thereby enabling comprehensive rehabilitative and assistive training. The exoskeleton robot is designed based on the anatomical structure of the human lumbar spine and the distribution of lumbar muscle groups, capable of providing assistance and support to the human waist. The upper and lower separated lumbar restraints, designed in accordance with ergonomics, feature a shape that conforms to the lumbar curve, allowing for better adaptation to the human lumbar region. Additionally, the adjustable upper and lower lumbar restraints are equipped with evenly distributed connection holes, which can be fixed on the waist rail according to the waist sizes of different populations, thus meeting the wearability requirements.

### 2.2. VSPAM

The shrinkable PAM for VSPAM is designed with a triple-chamber structure, consisting of three sets of pneumatic artificial muscles. These muscles are composed of flame-retardant nylon PET mesh tubing and highly elastic latex hoses, which are securely locked with self-locking nylon cable ties to prevent leakage. Without high-pressure gas and load, its maximum length is 45 cm. For each chamber, the highly elastic latex hose has a length of 40 cm, an outer diameter of 12 mm, and an inner diameter of 8 mm. The two end caps are 3D-printed using ordinary white resin, each featuring three protruding ports embedded in the highly elastic latex hose. Each port has a length of 3 cm and an outer diameter of 10 mm; the end cap itself has a length of 2.5 cm. One of the end caps is closed, while the other is equipped with three 6 mm air tubes, through which high-pressure compressed air can be injected into each air chamber of the shrinkable PAM. As the applied pressure increases, the length of the shrinkable VSPAM decreases until it reaches its maximum energy state, which occurs at a braiding angle of 54.7°. The effective length of the highly elastic latex hose involved in the shrinkage of a single air chamber is 34 cm.

The shrinkable PAM is constructed using the same type of braided mesh tubing and highly elastic latex hose as the contraction PAM. However, the highly elastic latex hose employed here has a static outer diameter four times that of the contraction PAM (48 mm) and a length of 40.5 cm. The two end caps of the stretch PAM are identical to those used in the contraction PAM, with each end cap maintaining effective contact with the highly elastic latex hose over a length of 2.5 cm. Consequently, the effective length of the highly elastic latex hose involved in the stretch PAM is 35.5 cm. Since the braided mesh tubing is significantly longer than the highly elastic latex hose, it needs to be axially compressed to match the length of the latter. This results in the static braided mesh tubing of the stretch PAM having a braiding angle greater than 54.7°, causing it to extend in length when subjected to compression.

**Figure 2:**
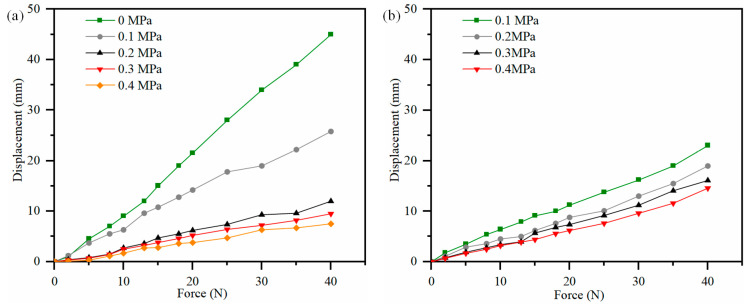
VSPAM mechanical property experiment. (**a**) Shrinkage VSPAM length varies with additional loading. (**b**) Length of stretched VSPAM varies with additional loading.

To calculate the stiffness of the stretched PAM, the experiment was repeated four times under five supply air pressures (0 MPa, 0.1 MPa, 0.2 MPa, 0.3 MPa, and 0.4 MPa), with the average value taken. The experimental results regarding the mechanical properties of the PAM are presented in Figure 2. Specifically, Figure 2a shows the experimental results of how the PAM length varies with different additional loads under a specific inflation pressure, while Figure 2b illustrates the variation in PAM stiffness as the supply air pressure increases. It is evident from the above figures that the contracted PAM exhibits greater stiffness than the stretched PAM. Experiments were also conducted to investigate the relationship between internal pressure and PAM length under no-load conditions. The results confirmed that the maximum length of the stretched VSPAM could reach 45 cm at a pressure of 0.4 MPa, which means its effective length was 13% longer than that in its natural resting state (without pressure application). The kinematic modeling of VSPAM is presented in the Supplementary Materials.

### 2.3. Control

The control system is divided into three layers: the perception layer, the conversion layer, and the execution layer. The perception layer primarily receives signals from various mechanical sensors and recognizes human movement intentions via an intention recognition algorithm. At the conversion layer, the Parameter Optimal Iterative Learning Control (POILC) method is employed [17]. This method maps the generated motion intentions into corresponding force generation trajectories. Finally, the execution layer controls and drives the flexible exoskeleton according to the force generation trajectories. Therefore, human motion intention recognition plays a crucial role in the control system of the flexible lower limb exoskeleton.

**Figure 3:**
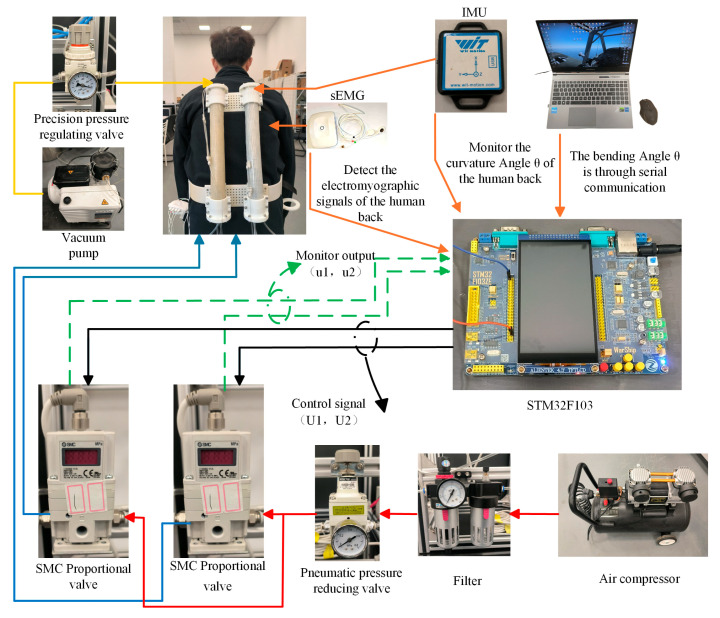
Experimental platform for lumbar spine-assisted exoskeleton robotic system.

To better control the assisting process of the lumbar spine-assisted exoskeleton robot and capture the real-time back bending angle of the human body, a nine-axis Bluetooth posture angle measurement sensor (BWT901CL) is installed at the end of the variable stiffness actuator. Real-time data transmission is achieved via Bluetooth, enabling monitoring of the movement direction and speed of the lumbar spine-assisted exoskeleton robot. Figure 3 presents the system integration framework of the lumbar spine-assisted exoskeleton robot. The hardware system of the robot comprises an air compressor, SMC electric proportional valves, analog converters, posture angle measurement sensors, a vacuum pump, a laptop computer, an STM32F103 minimum system board, and a 24 V power supply.

The solid red and blue lines represent the positive-pressure pneumatic circuits directing airflow to the variable-stiffness actuators within the exoskeleton robot, controlling the actuators to generate contraction force. The solid yellow line inside represents the negative-pressure pneumatic circuit, controlling changes in the actuator’s stiffness. The solid black line represents the electrical control signal from the proportional valve connected to the variable-stiffness actuator, while the green dashed line represents the output signal from the SMC proportional valve.

(a) Pneumatic Circuit Connections: Positive-Pressure Circuit: A high-pressure air compressor supplies gas. This gas passes through an oil-water separator/filter for purification, followed by a pneumatic pressure-reducing valve for pressure regulation. This prevents direct high-pressure exposure to the SMC proportional valves, protecting them. The setup employs two SMC proportional valves. Each valve generates three high-pressure gas streams; each connected to one of the three chambers within a variable-stiffness actuator. This drives the contraction force generation in the actuators for the lumbar-assist exoskeleton robot. Negative-Pressure Circuit: Within the lumbar-assist exoskeleton, the variable-stiffness module connects via a negative-pressure air fitting on the top base of the actuator to a precision pressure regulator. A vacuum pump provides a stable negative-pressure gas source to control the stiffness of the variable-stiffness module. The overall pneumatic flow paths are illustrated in Figure 3, where the red and blue lines represent the positive-pressure circuits, and the yellow line represents the negative-pressure circuit.

(b) Control Wiring Connections: Control Path: The signal cables from the two SMC proportional valves on the lumbar-assist exoskeleton connect to an STM32F103 microcontroller via a PWM-to-voltage converter module, enabling proportional control of the valve’s output pressure (shown as black lines in Figure 3). Feedback Path: An attitude sensor mounted on the end cap of the variable-stiffness actuator connects via Bluetooth to the STM32F103 microcontroller. This sensor is used to collect real-time data on the wearer’s lumbar flexion angle during rehabilitation training while wearing the exoskeleton. Additionally, each proportional valve has an output line connected to the STM32F103 via an analog acquisition module. This provides real-time feedback on the pressure values within each chamber of the variable-stiffness actuators, preventing potential damage caused by excessive pressure inflation.

### 2.4. Human–Machine Collaborative Movement

During active human torso rotation, the rotational kinetic energy of the pelvis transmits torque through the waist plate to drive the rotational work output of the vertebral body. Simultaneously, the contraction kinetic energy of the VSPAM injects supplementary energy, directly providing additional driving force for vertebral rotation. Algorithm 1 outlines the implementation workflow for human lumbar rotational motion.
**Algorithm 1** Rotational motion realizationsEMG sensor -> VGG-LSTM model: Collects back muscle electrical signals
VGG-LSTM model -->> Control decision: Output rotation intention probabilities (P_left, P_right)
Control decision ->> PAM1: If P_left > 0.8: The left PAM will contract by 70%
Control decision ->> PAM2: If P_right > 0.8: Right PAM dilates by 30%
Wearer’s pelvis -->> exoskeleton: Human body’s active rotation
Exoskeleton -->> Lumbar vertebra: Provides additional rotational torque

## 3. Multimodal Algorithm Recognition Model

**Figure 4:**
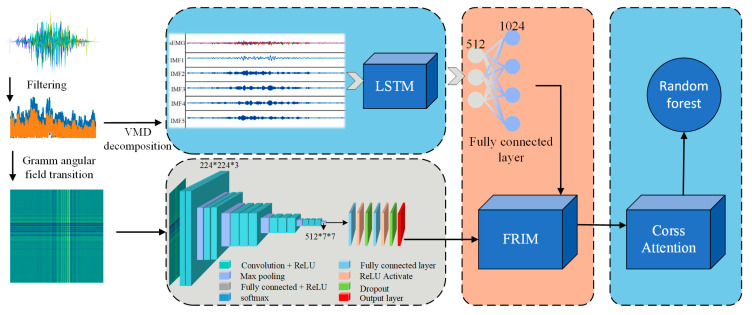
VGG-LSTM multimodal information fusion recognition algorithm.

The back movement intention recognition model in this study adopts a two-branch structure using raw sEMG data. Based on the waveform data, the one-dimensional sequence is converted into a two-dimensional Gram angle field image. One branch involves inputting the GAF into VGG16 for image feature extraction; the other branch processes the sEMG signal waveform through Variational Mode Decomposition (VMD) combined with the Welch method [18]. To obtain time-domain and frequency-domain features, these features are combined to form a flattened feature vector. Subsequently, sequence information is captured via LSTM, and the output of LSTM is converted to the same dimension as the image features through a fully connected layer. Finally, the image features and signal features are subjected to the Fine-grained Relationship Inference Module (FRIM) to form fused features. And then through the Cross Attention mechanism [19,20,21] used to enhance the representativeness of the features. Finally random forests are used for classification. Figure 4 shows the flowchart of the recognition algorithm.

### 3.1. Data Acquisition and Pre-Processing

**Figure 5:**
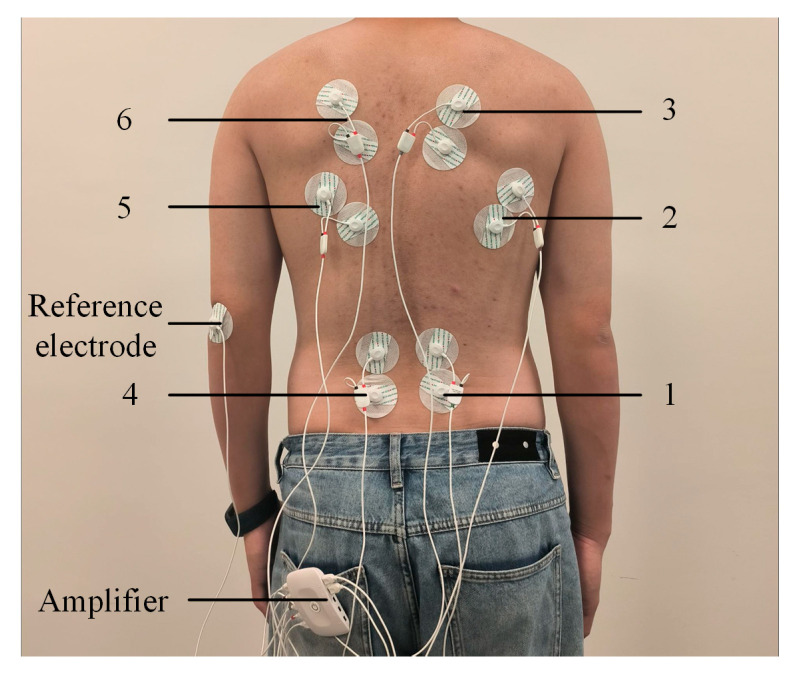
Position of electrode pads on the back of the volunteer.

The experiment was conducted by eight healthy participants, none of whom had a history of bone diseases. Their ages ranged from 20 to 35 years old, and their heights were between 160 cm and 185 cm. The experiment utilized 6 analog channels and 1 ground port, thus requiring 6 sEMG sensors and 13 electrode patches. Prior to the experiment, sEMG sensors 1 to 6 were attached to the human back as shown in Figure 5, with the reference electrode affixed to the wrist. To facilitate subsequent analysis and preprocessing of the experimental data, the 6 muscles and their corresponding 6 sEMG sensors are named as follows: sEMG sensors 1 and 4 correspond to the left and right erector spinae muscles of the back; sEMG sensors 2 and 5 correspond to the left and right latissimus dorsi muscles of the back; and sEMG sensors 3 and 6 correspond to the left and right trapezius muscles of the back.

**Figure 6:**
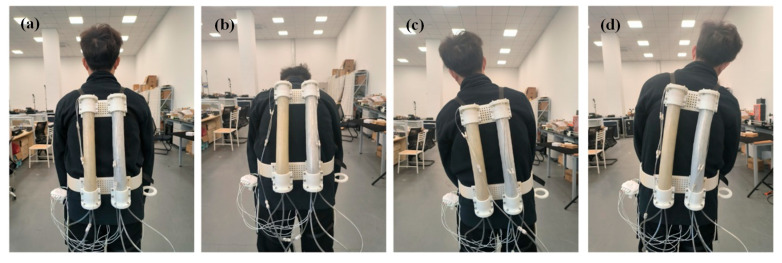
Volunteers wear lumbar spine to assist electromyographic signal acquisition experiments. (**a**) Upright condition. (**b**) Anterior lateral flexion. (**c**) Left lateral flexion. (**d**) Right side bending.

Prior to the experiment, the following preparations must be completed: First, each volunteer should ensure adequate sleep and avoid muscle fatigue on the day of data collection. Second, for sEMG signal acquisition on the human back, the indoor temperature must be strictly controlled within an appropriate range, avoiding extreme cold or heat to prevent interference with the sEMG signal. During the acquisition process, volunteers were first instructed to perform the following movements: Front Side Bending (FSB), Bend on the Left Side (BLS), Bend on the Right Side (BRS), Left Twist (LT), and Right Twist (RT). The start and end states of each movement were specified. Each volunteer repeated each movement 8 times, with an interval of 3–4 s between two consecutive repetitions of the same movement, totaling 5 sets. A 5 min rest was allowed between every two sets, and heart rate was monitored using a wearable bracelet to ensure that the volunteer’s back muscles under test were not fatigued before the start of each set of movements. The experiment for acquiring back sEMG signal without the volunteer wearing the lumbar spine-assisted exoskeleton is shown in Figure 6.

When acquiring signals via sEMG sensors, external factors such as ECG signals, ambient magnetic fields, and arm tremors can introduce noise into the collected sEMG signals. Therefore, it is necessary to denoise the raw acquired signals. sEMG signals result from the combined effects of superficial muscle electromyography and electrical activity in nerve trunks on the skin surface, with a wide frequency range; their main effective frequency components are distributed between 10 and 100 Hz. The wavelet threshold denoising method was employed to filter out high-frequency noise in the signals. Specifically, the original signals were subjected to 4-layer wavelet decomposition using the db2 wavelet basis function. The denoised sEMG data were then processed with a 4th-order Butterworth band-pass filter to suppress low-frequency drift and high-frequency noise, thereby improving the signal-to-noise ratio.

Active segment detection uses a simple moving average method to calculate the instantaneous energy of the data during action transitions:

(1) $$MAS_{t}=\frac{X_{t-n+1}+X_{t-n+2}+\cdots +X_{t}}{n}$$

where *MAS_t_* (moving average series) denotes the moving average at time t, *X_t_* denotes the value of the signal at time *i*, and *n* is the window size (i.e., the number of data points included) of the moving average. This formula obtains the moving average of the current time point by calculating the average of the last *n* data points. A suitable threshold is also set so that when the *MAS* value is greater than the threshold, the signal within the window is considered to be an active segment.

### 3.2. Principles of Related Algorithms

#### 3.2.1. sEMG GAF Model

Wang et al. [22] originally proposed a GAF algorithm, the algorithm encodes one-dimensional time series data into a kind of two-dimensional image. It is outlined as follows: time series *X* = {*X*_1_, *X*_2_, …, *X_n_*} The time series containing measured values is normalized to values between 0 and 1:

(2) $${\tilde{x}}_{i}=\frac{\left(x_{i}-max(X))+(x_{i}-min(X)\right)}{max(X)-min(X)},(i=1,2,\ldots n)$$

Next, the arccosine function is employed to convert the one-dimensional time series into a polar coordinate system, where the timestamp serves as the radius. This transformation is expressed in Equation (3):

(3) $$\left\{\begin{array}{c} \phi =\arccos({\tilde{x}}_{i}),\;0\leq {\tilde{x}}_{i}\leq 1,\;{\tilde{x}}_{i}\in \tilde{X}\\ r=\frac{t_{i}}{N},\;\;\;\;\;\;\;\;\;\;\;\;\;\;\;\;\;\;\;\;\;\;\;\;\;\;\;\;\;\;\;\;\;\;\;t_{i}\in N \end{array}\right.$$

where *t_i_* is the timestamp, *N* is a constant factor to adjust the polar range, and *Φ* is the angular cosine of each value in the time series. The coded mapping of Equation (3) has two important properties. First, it is bijective when cos(*φ*) is monotonic when *φ* ∈ [0, π]. Given a time series, the proposed mapping produces and only one result with a unique inverse function in the polar coordinate system. It provides a way to maintain time dependence as time increases as the position moves from the upper left to the lower right corner. GAF contains temporal correlation as *G*(i, ji j = k) represents the relative correlation by superimposing the directions with respect to the time interval *k*. The proposed mapping is a time-dependent mapping with a unique inverse function in the polar coordinate system.

Finally, after converting the scaled time series into values within the polar coordinate system, an angular perspective is adopted. This involves determining the correlation of the time series at different time intervals by considering the triangular sums or differences between each point. There are two distinct encoding methods for the GAF: The Gramian Angular Sum Field (GASF) and the Gramian Angular Difference Field (GADF), as expressed in Equation (4).

(4) $$\begin{array}{l} GASF=\left[\begin{array}{ccc} \cos(\phi_{1}+\phi_{1}) & \ldots & \cos(\phi_{1}+\phi_{n})\\ \vdots & \ddots & \vdots \\ \cos(\phi_{n}+\phi_{1}) & \ldots & \cos(\phi_{n}+\phi_{n}) \end{array}\right]\\ GADF=\left[\begin{array}{ccc} \sin(\phi_{1}-\phi_{1}) & \ldots & \sin(\phi_{1}-\phi_{n})\\ \vdots & \ddots & \vdots \\ \sin(\phi_{n}-\phi_{1}) & \ldots & \sin(\phi_{n}-\phi_{n}) \end{array}\right] \end{array}$$

**Figure 7:**
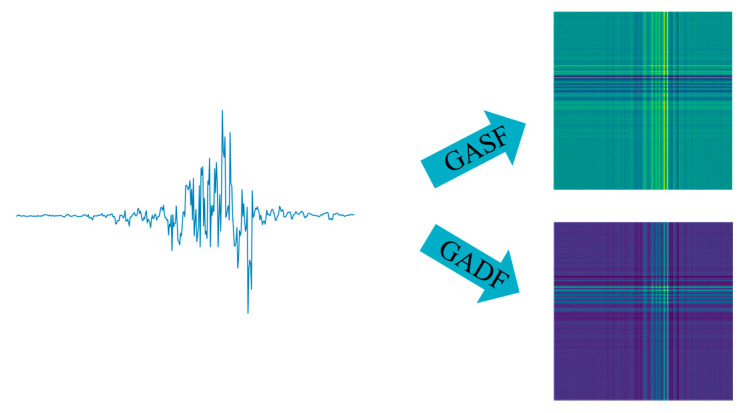
GADF\GASF mapping map.

Using the GAF method can maintain the time series characteristics of the signal, because the polar transformed time series is input from the upper left corner to the lower right corner along with the increase in time, so that the converted image can maximally retain the characteristics that the original signal has, which can effectively help us to identify the type of faults using the convolutional neural network. Figure 7 depicts the results of converting time series signals into images using two different GAF coding techniques.

#### 3.2.2. FRIM

**Figure 8:**
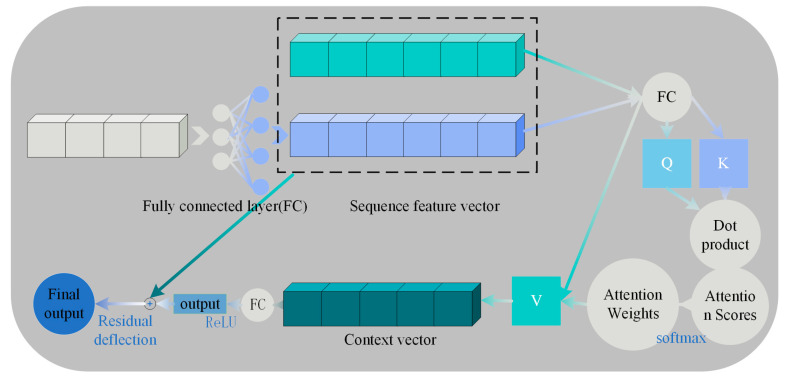
Fine-grained relational reasoning.

A FRIM based on a self-attention mechanism is implemented. The self-attention mechanism allows a module to dynamically adjust its attention as it processes the input. The module can dynamically assign different attention weights according to different parts of the input data, thus capturing the fine-grained relationships between the data more accurately. The specific flow is shown in Figure 8.

In the self-attentive mechanism, the representation of the input sequence generates context-aware features through a series of linear projections with interactive computations. The mathematical process can be formally described as follows: Given an input sequence *X*, a query matrix *Q* is generated by linear projection through three independently learnable weight matrices *W_Q_*, *W_K_* and *W_V_*, key matrix *K* and value matrix *V*:

(5) $$Q=W_{Q}\cdot x,\;K=W_{K}\cdot x,\;V=W_{V}\cdot x$$

The transpose of the query matrix to the key matrix computes the unnormalized attention scores via the dot product operation, and the scaling factor $\sqrt{d_{k}}$ adjustment of numerical stability:

(6) $$\mathrm{AttentionScores}=Q\cdot K^{T}/\sqrt{d_{k}}$$

Attention weights are obtained by softmax normalizing the attention scores. The softmax function is able to convert each element in a vector to a value between 0 and 1 and the sum of these values is 1.

(7) AttentionWeights=softmax(Attention Scores)

The attention weights are applied to the values to obtain the weighted values (i.e., the context vector).

(8) Context=Attention Weights⋅V

This operation ensures that the attention weights satisfy non-negativity ($a_{ij}$ ≥ 0) with normalization ($\sum_{j}a_{ij}$ = 1), characterizing the strength of dependence between positions within the sequence.

Finally, the weighted values (context vectors) are passed through a linear layer for feature fusion and the ReLU activation function is applied. At the same time, the fused features are summed with the original input *x* in order to achieve residual connectivity.

(9) Output=LayerNorm(ReLU(OutFeatureFusion)+X)

Among them, Layer Normalization is used to mitigate the gradient vanishing problem and ReLU activation function introduces nonlinearity. Residual concatenation then preserves the original input information to optimize deep training. Out Feature Fusion represents the result after feature fusion.

## 4. Experimental Results and Analysis

The experiments of this study were run in Python 3.10 environment with a computer configuration of Window 11 operating system, 32 GB of operating memory, AMD Ryzen 7 7840H with Radeon 780M Graphics processor, and NVIDIA GeForce RTX 4060 Laptop GPU as the graphics card model.

### 4.1. Movement Prediction Experiment

This study constructed a dataset containing 16,000 samples, where each sample corresponds to a specific action and consists of one image paired with its associated textual description. The dataset is divided into a training set and a test set at an approximate ratio of 80% to 20%. For the VGG-LSTM network, the initial learning rate is set to 0.0001, with a learning rate decay factor of 0.1 and a decay step size of 10. The maximum number of iterations is set to 50, and the batch size is 40. The network employs the Adam adaptive optimization algorithm, with the cross-entropy loss function selected as the loss function. During each training session, 50% of the output features are randomly discarded to mitigate overfitting. The model demonstrating the best performance is saved during the training process.

To evaluate the impact of key components of the model on the overall system performance, this study conducts ablation experiments. By gradually removing specific components of the model, it observes how such changes affect the model’s performance, thereby assessing the model’s robustness and stability.

**Figure 9:**
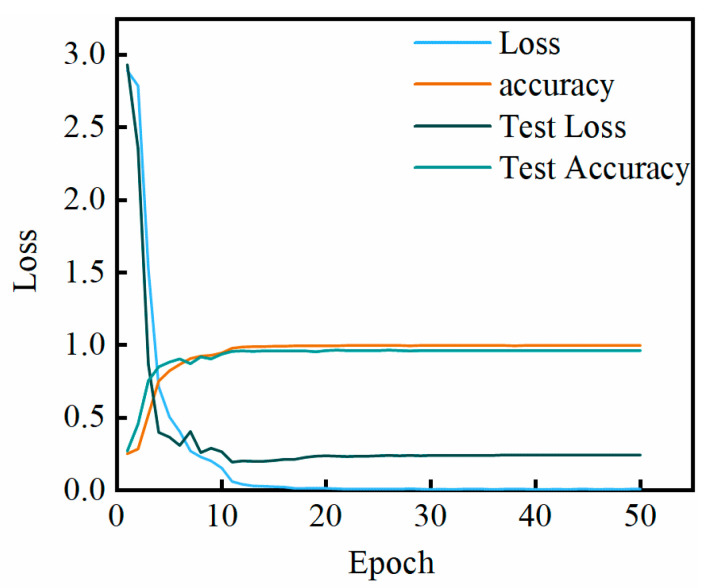
Accuracy of multimodal VGG-LSTM with loss variation.

A comparison was made among four model groups: “VGG only”, “LSTM only”, “VGG+LSTM (without CAM)”, and “VGG+LSTM+CAM+FRIM”, with the following findings: VGG only: It performs well in static action classification, but the accuracy of dynamic intention prediction declines. LSTM only: It is sensitive to noise, and the lack of frequency domain information leads to classification confusion. VGG+LSTM (without CAM): Feature splicing results in overfitting, and the accuracy of the validation set fluctuates significantly. VGG+LSTM+CAM+FRIM: The model’s robustness and accuracy are significantly improved through attentional filtering. Figure 9 shows the changes in accuracy and loss of the VGG-LSTM algorithm.

### 4.2. Comparative Experiments

**Figure 10:**
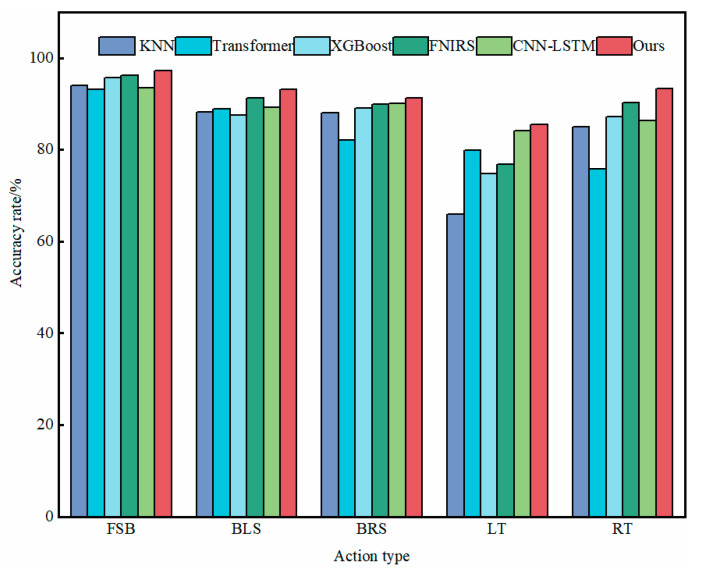
Classifier comparison experiment.

To evaluate the performance of the proposed VGG-LSTM model in sEMG signal recognition, we conducted a comparative analysis with multiple benchmark classifiers, encompassing both classical and state-of-the-art algorithms. Specifically, the selected classifiers include K-Nearest Neighbors (KNN), eXtreme Gradient Boosting (XGBoost), Transformer [23], Functional Near-Infrared Spectroscopy (FNIRS)-based methods [24], and CNN-LSTM [15]. The comparative results are presented in Figure 10.

### 4.3. Experiment on the Effectiveness of Lumbar Spine Assisted Robot

**Figure 11:**
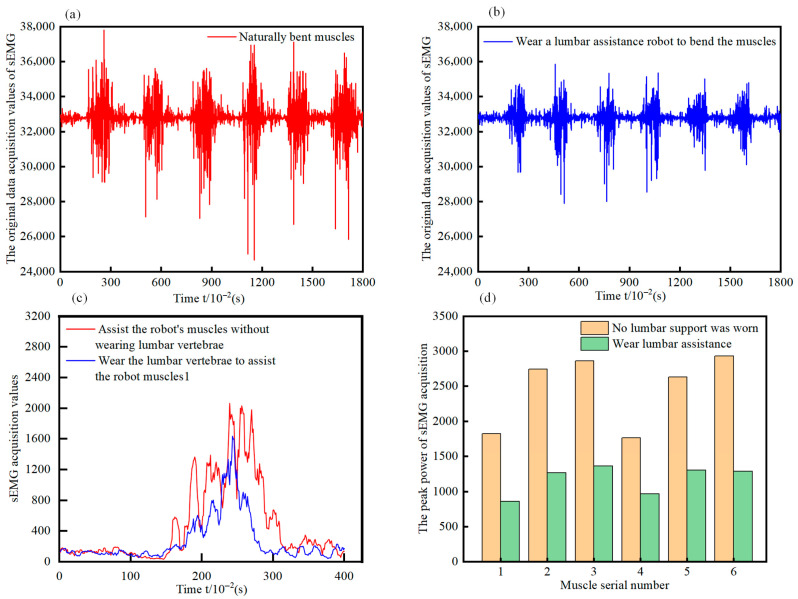
Experiment on the effectiveness of lumbar spine assisted robot (**a**) Unworn sEMG signal preprocessing results. (**b**) Wearable EMG signal pre-processing results. (**c**) Time-series comparison of sEMG signal waveforms. (**d**) Temporal peak power values in sEMG recordings.

Raw data were acquired based on human back sEMG signals. The sEMG signals were selected and preprocessed, and the preprocessing results are shown in Figure 11a,b, which illustrate the sEMG signals when wearing and not wearing the exoskeleton. For anterior lateral flexion movements, the sEMG signals exhibit significant changes. The intensity of the collected sEMG signals on the back can reflect the magnitude of muscle movement, i.e., to a certain extent, the degree of muscle contraction or relaxation. After collecting relevant data using the sEMG sensor and performing corresponding data processing, the collected values of back sEMG signals from the six back muscles before and after assistance can be obtained. During human back-bending movements, the amplifier captures changes in sEMG intensity and converts them into corresponding values. These values are quantified using a power value w, which is used to visualize the strength of the sEMG signal.

To more precisely evaluate the force-saving effect on the human back muscle groups after using the lumbar spine-assisted robot, a comparative analysis was performed on sEMG signals recorded with and without the exoskeleton. The preprocessed sEMG signals corresponding to the left and right symmetrical erector spinae, latissimus dorsi, and trapezius muscles—both before and after assistance—were averaged. Subsequently, this average value was subtracted from the data of each experimental trial, and the absolute values were calculated. The duration of a single movement cycle was standardized to 4 s, thereby enabling a clear visualization of the pronounced assistive effect, as illustrated in Figure 11c, which depicts the comparative changes in sEMG signals for Volunteer A with and without the exoskeleton. From the comparative graph, it is evident that during the anterior lateral bending motion, the power value of the sEMG signal (*w*) is significantly lower when the lumbar spine-assisted robot is worn compared to when it is not.

As shown in Figure 11d, regarding the peak power of sEMG signals, after wearing the lumbar spine-assisted exoskeleton robot, the maximum power values of the six muscles during human anterior lateral bending and left-right bending movements have been significantly reduced. To quantify the assistance provided by the lumbar robot to the user, the %*MVC* standardization method was used. The subjects were required to perform the maximum isometric contraction (*MVC*) in a specific posture on the front side of the back, and the Root Mean Square (*RMS*) peak value of sEMG for each muscle was recorded three times. The maximum value was taken as the benchmark, and finally, the %*MVC* of sEMG during the movement was calculated:

(10) $$\%MVC=\frac{\mathrm{Action}\;\mathrm{period}\;sEMG(RMS)}{MVC\;\mathrm{period}\;sEMG(RMS)}\;\times \;100\%$$

The study found that the muscle activation level of the users decreased by 40% ± 5%. This can provide assistance to the human waist, indicating that the designed lumbar assistive exoskeleton robot can provide the required contraction force.

A paired *t*-test was used to compare the sEMG power values (data of the latissimus dorsi muscle) of the same volunteer before and after wearing the device. Hypothesis test: H0 (null hypothesis) is that there is no difference in the means before and after wearing; H1 (alternative hypothesis) is that there is a difference. The t-statistic can be estimated as:

(11) $$t=\frac{\bar{d}}{s_{d}/\sqrt{n}}$$

Here, $\bar{d}$ is the mean difference, and *s_d_* is the standard deviation of the difference. If the result *p* < 0.05, then the null hypothesis H0 is rejected, and it is confirmed that the reduction is significant. $\bar{d}$: The average reduction is 40% (i.e., the mean effect size is 40%), *s_d_*: ±5% (i.e., the standard deviation is 5%), sample size (*n*): 8. The calculated t-value is approximately 22.627, with a sample size *n* = 8, and the corresponding degrees of freedom *df* = *n* − 1 = 7. Through the t-distribution table, it can be found that the *p*-value corresponding to this t-value is much less than 0.05. Therefore, it can be confirmed that “an average reduction of 40%” is significant.

### 4.4. Real-Time Latency Testing Experiment

The end-to-end latency of the system is 110 ± 15 ms (from the activation of the erector spinae muscle to the output of the vertebral body assistive moment), which is significantly lower than the period of the human body’s autonomous trunk rotation movement (300–500 ms), meeting the real-time requirements of the waist assistance scenario. The actuators account for more than 40% of the latency distribution. Table 1 is real-time latency testing experiment. In the future, performance can be further improved through high-response VSPAM optimization.

## 5. Conclusions

This study applies variable-stiffness robotic technology to the design of a lumbar rehabilitation exoskeleton robot. It addresses the challenges of providing multi-dimensional assistance and support to the lower back during rehabilitation exercises while reducing the risk of spinal injury. Simultaneously, it proposes a novel approach for upper limb motion intention recognition based on multi-modal information fusion. Compared to traditional sEMG-based action recognition research, this method incorporates real-time motion intention recognition. To validate the algorithm’s effectiveness, a VGG-LSTM recognition framework was constructed and tested on an sEMG signal dataset for action recognition. Comparative experimental results demonstrate that the VGG-LSTM algorithm exhibits excellent performance. Furthermore, the VGG-LSTM model meets the real-time requirements for human motion intention recognition, achieving an average recognition accuracy of above 80% during action prediction. This technology provides safe, efficient, compliant, and natural assistance and rehabilitation equipment for elderly individuals and patients with chronic low back conditions. It holds significant promise for alleviating the rehabilitation and care burdens associated with an aging population and chronic back diseases. At the same time, it can be effectively used by factory workers, warehouse pickers, and manual handlers performing operations such as bending, lifting, and material handling in fixed-location workplaces. It provides protection for the lower back, back, and leg muscles, reducing occupational strain. which is of great social significance. In this study, only the back movement sEMG signal dataset is tested, and the next step is to collect sEMG signals for gesture and lower limb rehabilitation exoskeleton robots, and to incorporate force, posture, and other sensors to obtain a more reliable recognition of human movement intention.

## Figures and Tables

**Table 1:** Real-Time Latency Testing Experiment.

Delaying Stage	Measurement Method	Mean ± Standard Deviation
sEMG Collection delay	Simulated impulse response	12 ± 3 ms
Model inference delay	Real-time inference within a 200 ms window	28 ± 5 ms
VSPAM contraction response delay	10–90% travel time test	45 ± 8 ms
End-to-end closed-loop delay	Electromyography triggering → Spinal movement capture	110 ± 15 ms

## Data Availability

DOI: https://doi.org/10.57760/sciencedb.j00003.00040.

## Supplementary Materials

The following supporting information can be downloaded at: https://www.mdpi.com/article/10.3390/biomimetics10090556/s1, Figure S1. The general geometry of pneumatic artificial muscle; Figure S2. Kinematics of VSPAM: (a) General geometry of contraction and extensor PAMs; (b) Angle and number of turns of woven mesh for contraction and extensor PAMs; (c) Length relationship between contraction and extensor PAMs; (d) Design of VSPAM.
