# Supplementary material for: Motion Intention Prediction for Lumbar Exoskeletons Based on Attention-Enhanced sEMG Inference
Source: Biomimetics (Basel). 2025 Aug 22;10(9):556. doi: 10.3390/biomimetics10090556 (PMC12467838; doi:10.3390/biomimetics10090556)
Supplement: Supplementary file 1 [file biomimetics-10-00556-s001.zip › biomimetics-3758579-supplementary.pdf]

Figure S1 shows the general geometry of a pneumatic artificial muscle. Assuming the middle section is a perfect cylinder, the pneumatic artificial muscle has length L, diameter d, and θ denotes the braiding angle between a single braided thread and the central axis. The length of a single braided thread is L_b. The braided sleeve wraps n turns around the highly elastic latex tube inside the muscle. The flame-retardant nylon PET braided sleeve is longer than the highly elastic latex tube, requiring compression by an angle β to match the latex tube's length.

In VSPAM, the difference between extending-PAM and contracting-PAM lies in the resting length of their latex tubes. Specifically, the highly elastic latex tube in contracting-PAM has a significantly longer resting length than that in extending-PAM. According to Figure S1, given the initial length L and diameter D of the pneumatic artificial muscle:

$$L = b \cos\beta \qquad \text{S(1)}$$

$$D = \frac{b \sin\beta}{n\pi} \qquad \text{S(2)}$$

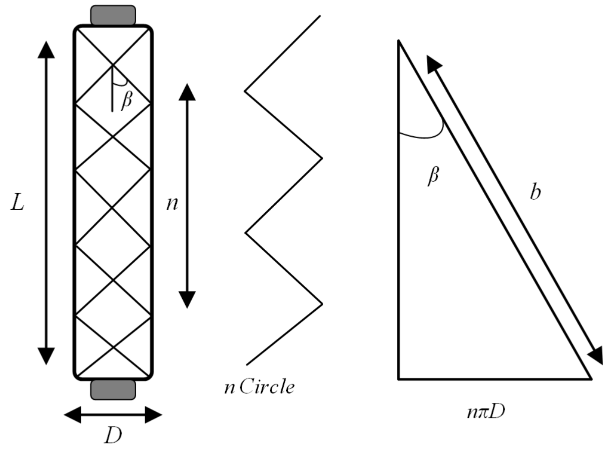

Figure. S1. The general geometry of pneumatic artificial muscle

Assuming that the middle section of the pneumatic artificial muscle is a cylinder, the volume of the actuator would be:

$$V = \frac{D^2 L\pi}{4} \qquad \text{S(3)}$$

The analysis of the variable stiffness pneumatic artificial muscle (VSPAM) designed in this paper is based on the following assumptions: there is no friction or elastic force between the flame-retardant nylon PET braided tube and the high elasticity latex hose, between the nylon wires of the flame-retardant nylon PET braided tube, and between the stretching pneumatic artificial muscle and the contracting pneumatic artificial muscle. Figure S2 illustrates the geometric kinematics analysis of VSPAM. VSPAM uses the same contracting PAM and stretching PAM (discussed in the

"Contracting PAM" and "Stretching PAM" sections above). The geometric relationship between these two pneumatic artificial muscles is as follows: the inner diameter of the high elasticity latex hose of the stretching PAM is four times that of the contracting PAM (both have the same rubber hose type). According to the above experimental results, the contracting PAM is 13% longer than the stretching PAM, as shown in Figure S2(c). Therefore, at the static length of VSPAM, the contracting PAM will bend or compress inside the stretching PAM to match the length of the stretching PAM, as shown in Figure S2(d).

$$L_C = 1.13 L_E \tag{S(4)}$$

$$L_E = \frac{1}{1.13} L_C \tag{S(5)}$$

where L_C is the length of the contractile PAM, and L_E is the length of the extensible PAM.

The length of the extensible PAM's braided mesh corresponds to the initial length of the VSPAM when no high-pressure gas is injected. The inner diameter of the high-elasticity latex tube in the extensible PAM is four times larger than that in the contractile PAM. Therefore, the relationship between the resting diameters of their high-elasticity latex tubes is as follows:

$$n_C = 1.13 n_E \tag{S(6)}$$

$$4 b_C = 1.13 b_E \tag{S(7)}$$

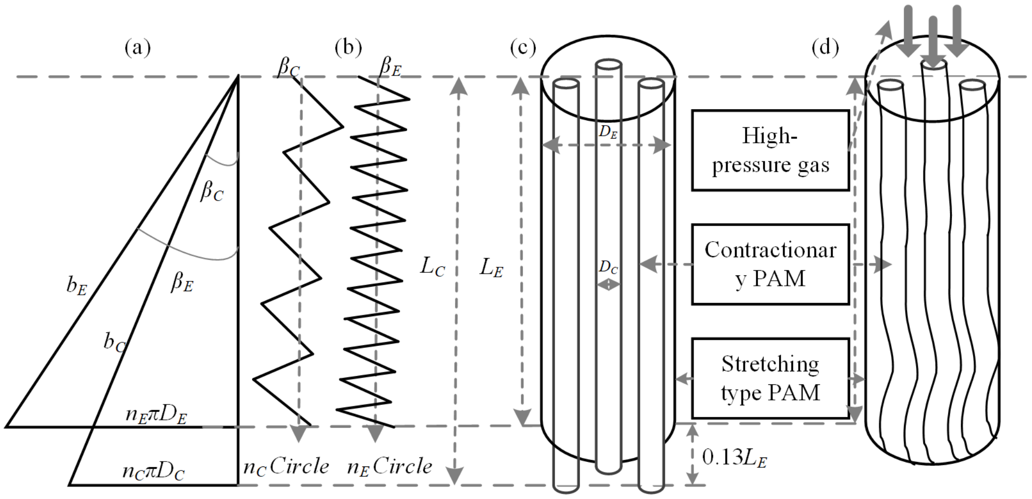

Figure. S2. Kinematics of VSPAM: (a) General geometry of contraction and extensor PAMs); (b) Angle and number of turns of woven mesh for contraction and extensor PAMs; (c) Length relationship between contraction and extensor PAMs; (d) Design of VSPAM

where n_C represents the number of braided mesh turns for the contractile PAM, n_E represents the number of braided mesh turns for the extensible PAM, b_C denotes the

single-thread length of the braided mesh for the contractile PAM, and b_E denotes the single-thread length of the braided mesh for the extensible PAM.

According to the geometric parameters of the contractile PAM and extensible PAM shown in Figure S2:

$$L_C = b_C \cos \beta_C \quad \text{S(8)}$$

$$L_E = b_E \cos \beta_E \quad \text{S(9)}$$

$$D_C = \frac{b_C \sin \beta_C}{n_C \pi} \quad \text{S(10)}$$

$$D_E = \frac{b_E \sin \beta_E}{n_E \pi} \quad \text{S(11)}$$

$$V_C = \frac{\pi D_C{}^2 L_C}{4} \quad \text{S(12)}$$

$$V_E = \frac{\pi D_E{}^2 L_E}{4} \quad \text{S(13)}$$

where β_C is the angle between the braided thread in the flame-retardant nylon mesh sleeve and the central axis of the high-elasticity latex tube in the contractile PAM, β_E is the angle between the braided thread in the flame-retardant nylon mesh sleeve and the central axis of the high-elasticity latex tube in the extensible PAM, D_C is the diameter of the contractile PAM, D_E is the diameter of the extensible PAM, V_C is the volume of the contractile PAM, and V_E is the volume of the extensible PAM.

To simulate the output force of the Variable-Stiffness Pneumatic Artificial Muscle (VSPAM), which combines both extension and contraction functions, a mathematical model of the PAM's output force is derived based on its cylindrical geometry as follows:

$$F = -P^1 \frac{dV}{dL} \quad \text{S(14)}$$

where P^1 is the pressure difference between the inside and outside of the PAM.

In the VSPAM system, the relative air pressure within the high-elasticity latex tube of the contractile PAM is influenced by the internal pressure of the extensible PAM's latex tube (i.e., higher pressure in the extensible PAM reduces the relative pressure in the contractile PAM). Therefore:

$$P^1 = (P_C - P_E) \quad \text{S(15)}$$

where P_C is the air pressure of the contractile PAM, and P_E is the air pressure of the

extensible PAM.

By substituting Equations S(8), S(12), and S(15) into Equation S(14), the contraction force F_C generated by the contractile PAM is obtained:

$$F_C = -(P_C - P_E)\frac{dV_C}{dL_C} \tag{S(16)}$$

Differentiating Equation S(16) with respect to θ_C yields:

$$F_C = -(P_C - P_E)\frac{dV_C/d\beta}{dL_C/d\beta} = \frac{b_C{}^2(P_C - P_E)}{4\pi n_C{}^2}(3\cos^2\beta_C - 1) \tag{S(17)}$$

The extensible PAM is affected by the volume of the contractile PAM. In practice, the contractile PAMs arranged in an equilateral triangular configuration occupy the central hollow cylindrical portion of the extensible PAM. This means the actual volume of the extensible PAM must account for the displacement by the contractile PAMs. Therefore, the force F_S generated by the extensible PAM will be:

$$F_S = P_E\frac{dV_S}{dL_E} = P_E\frac{dV_E - 3dV_C}{dL_E} \tag{S(18)}$$

where V_S represents the actual cylindrical volume of the extensible PAM.

Substituting the relationship between the contractile PAM and extensible PAM from Equation S(4) into Equation S(18) yields:

$$F_S = P_E\left(\frac{dV_E}{dL_E} - 1.13\frac{dV_C}{dL_C}\right) \tag{S(19)}$$

Differentiating Equation S(19) with respect to θ_E gives:

$$F_S = P_E\left(\frac{dV_E/d\theta_E}{dL_E/d\theta_E} - 1.13\frac{dV_C/d\theta_C}{dL_C/d\theta_C}\right) = P_E\left(1.13\frac{b_C{}^2(3\cos^2\theta_C - 1)}{4\pi n_C{}^2} - \frac{b_E{}^2(3\cos^2\theta_E - 1)}{4\pi n_E{}^2}\right)$$

(20)

Substituting Equations S(6) and S(7) into Equation S(20) and simplifying yields:

$$F_S = \frac{b_C{}^2 P_E}{4\pi n_C{}^2}\left[1.13\left(3\cos^2\theta_C - 1\right) - 16\left(3\cos^2\theta_E - 1\right)\right] \tag{S(21)}$$

In the VSPAM system, the contraction force F_C generated by the contractile PAM and the extension force F_S produced by the extensible PAM are combined to yield a resultant force, which represents the telescopic force generated by the VSPAM:

$$F = F_C - F_S = \frac{{b_C}^2}{4\pi {n_C}^2}\begin{bmatrix} P_C\left(3\cos^2\theta_C - 1\right) - \\ 10.08P_E\left(3\cos^2\theta_C - 1\right) + \\ 16P_E\left(3\cos^2\theta_E - 1\right) \end{bmatrix} \tag{S(22)}$$

when $F > 0$, it represents the contraction force generated by the VSPAM; when $F < 0$, it indicates the extension force produced by the VSPAM.
